# Specnuezhenide Ameliorates Age-Related Hepatic Lipid Accumulation via Modulating Bile Acid Homeostasis and Gut Microbiota in D-Galactose-Induced Mice

**DOI:** 10.3390/metabo13080960

**Published:** 2023-08-18

**Authors:** Xuehui Deng, Bingfeng Lin, Fang Wang, Pingcui Xu, Nani Wang

**Affiliations:** 1 School of Pharmacy, Zhejiang Chinese Medical University, Hangzhou 310007, China; 202111113611026@zcmu.edu.cn (X.D.);; 2 Department of Medicine, Zhejiang Academy of Traditional Chinese Medicine, Hangzhou 310007, China; bingfengl1995@163.com (B.L.);

**Keywords:** Specnuezhenide, lipid accumulation, bile acids, gut microbiota

## Abstract

Age-related hepatic lipid accumulation has become a major health problem in the elderly population. Specnuezhenide (SPN) is a major active iridoid glycoside from an edible herb *Fructus Ligustri Lucidi*, which is commonly used for preventing age-related diseases. However, the beneficial effects of SPN on age-related liver injury remain unknown. This study aimed to reveal the effect of SPN on age-related hepatic lipid accumulation and the underlying mechanism. D-galactose (D-gal)-induced aging mice were treated with vehicle or SPN for 12 weeks. Treatment of SPN decreased lipid accumulation and inflammation in the liver of D-gal–induced mice. Untargeted and targeted metabolomics showed that the SPN could regulate the bile acid (BA) synthesis pathway and restore the BA compositions in serum, livers, and feces of the D-gal–induced mice. Furthermore, SPN enhanced the protein and mRNA levels of hepatic BAs synthesis enzymes cytochrome P45027A1, cytochrome P4507A1, cytochrome P4507B1, and cytochrome P4508B1. Meanwhile, SPN alleviated D-gal-induced gut dysbiosis and reversed the proportions of microbes associated with bile salt hydrolase activity, including *Lactobacillus*, *Ruminiclostridium*, and *Butyrivibrio*. Our study revealed that SPN attenuated age-related hepatic lipid accumulation by improving BA profiles via modulating hepatic BA synthesis enzymes and gut microbiota.

## 1. Introduction

As human life expectancy keeps increasing, aging-related health deterioration has been a growing challenge for clinical practices. Nonalcoholic fatty liver disease (NAFLD) is a major cause of chronic liver disease and has been prevalent in the elderly population [1]. NLFLD is a liver metabolic disorder that results from increased total cholesterol (TC) and total triglyceride (TG) accumulation [2]. Bile acids (BAs) are hepatic metabolites and have a close relationship with the pathophysiology of NAFLD. BAs have a well-established role in the regulation of cholesterol and lipid metabolism [3]. Disordered BA homeostasis commonly accompanies aging [4], which increases the risk of aging-related hepatic lipid accumulation [5]. Clinical studies proved that altered BA profiles underlie the development of NAFLD. Dietary BA supplementation can ameliorate high dietary lipid-induced liver injury by reducing lipid accumulation and improving liver injury [6]. Thus, targeting BA metabolism has the potential to prevent hepatic lipid accumulation and NAFLD.

Specnuezhenide (SPN), an iridoid glycoside, is the main component of *Fructus Ligustri Lucidi*, which is an edible herb commonly used to prevent aging-related diseases [7]. The extract of *Fructus Ligustri Lucidi* could inhibit hepatic injury by carbon tetrachloride–induced mice [8], regulated fat balance in ovariectomized mice [9], and preserved bone mass in D-galactose (D-gal)/sodium nitrite-induced mice [10]. Iridoid glycosides and SPN could reduce the development of liver injury in concanavalin A–induced mice [11] and carbon tetrachloride–induced mice [12], respectively. However, it is not clear whether SPN can attenuate age-related hepatic lipid accumulation and regulate BA homeostasis.

The current study aimed to describe the effect of SPN on age-related hepatic lipid accumulation and determine the mechanism of SPN with BA homeostasis in D-gal–induced mice. We established that SPN could reduce D-gal–induced hepatic lipid accumulation and regulate BA homeostasis. Moreover, SPN improved BA profiles by increasing the expressions of BA synthesis enzymes in the liver and modulating the gut microbiota community structure of D-gal–induced mice.

## 2. Materials and Methods

### 2.1. Mice and Treatment

ICR mice (20 ± 2 g, 8 weeks, male) were purchased from Hangzhou Medical College (Hangzhou, China). The animal basal diet (#P1101F-25; Slacom, Shanghai, China) contained protein (20.5%), fat (4.0%), calcium (1.0–1.8%), lysine (1.3%), phosphorus (0.6–1.2%), methionine + cystinol (0.8%), and sodium chloride (0.4%). The experiments were performed in accordance with the NIH Guide for the Care and Use of Laboratory Animals (NIH Publication No. 80–23; revised 1978) under the Guidelines for Animal Experiments of the Zhejiang Academy of Traditional Chinese Medicine (Approval No. 2021-001). The protocol was approved by the Institutional Animal Ethics Committee. SPN (#102728, purity ≥ 98%; Yongjian Pharmaceutical, Taizhou, China) was dissolved in sterile saline for oral gavage (i.g.). Requirements for natural product pharmacological research were taken into account [13,14]. The mice were randomly divided into a normal control group (CON, sterile saline daily i.g. + subcutaneous injection (s.q.) of sterile saline daily, *n* = 10), model group (MOD, sterile saline daily i.g. + 150 mg/kg/d D-gal s.q. daily, *n* = 10), SPN with low dosage (SPN-L, 5 mg/kg/d SPN i.g. + 150 mg/kg/d D-gal s.q. daily, *n* = 10), and SPN with high dosage (SPN-H, 10 mg/kg/d SPN i.g. + 150 mg/kg/d D-gal s.q. daily, *n* = 10) for three months. The number of animals per group was determined based on previous reports [15]. The dose and duration of SPN were used according to previous reports [16,17]. After a 12-week treatment, mice serum, feces, and liver tissues were collected. The liver index was calculated according to the following equation: liver index = liver weight/body weight × 100%.

### 2.2. Haematoxylin-Eosin Staining (H&E) and Immunohistochemistry (IHC)

Liver samples were fixed in 4% paraformaldehyde, embedded in paraffin, and sectioned at 4 μm thickness. Tissue sections were stained with H&E staining. For the IHC analysis, the sections were incubated with primary antibodies against anti-cytochrome P4507A1 (CYP7A1), anti-cytochrome P4507B1 (CYP7B1), anti-cytochrome P4508B1 (CYP8B1), and anti-cytochrome P45027A1 (CYP27A1).

### 2.3. Analysis of TC, TG, Low-Density Lipoprotein Cholesterol (LDL-C), and High-Density Lipoprotein Cholesterol (HDL-C)

The commercially available kits were used to determine the levels of TC (#A111-1-1; Nanjing Jiancheng Bioengineering Institute, Nanjing, China), TG (#A110-2-1; Jiancheng Bioengineering), LDL-C (#A113-1-1; Jiancheng Bioengineering), and HDL-C (#A112-1-1; Jiancheng Bioengineering) in the liver. The absorbance was obtained using a microplate reader (Spectra MAX 190; Molecular Devices, Silicon Valley, CA, USA).

### 2.4. Untargeted Metabolomics Analysis

A total of 100 μL of serum was mixed with 1 mL of acetonitrile/methanol (acetonitrile:methanol = 4:1, *v*/*v*) containing an internal standard (L-2-chlorophenylalanine, 2 mg/L), homogenized, sonicated, and centrifuged. A total of 800 μL of supernatant was lyophilized. The residue was redissolved in acetonitrile/methanol/water (200 μL, acetonitrile:methanol:water = 2:2:1, *v*/*v*) with formic acid (0.1%). An ultraperformance liquid chromatography (UPLC; ACQUITY, Waters, Milford, MA, USA) coupled to a mass spectrometry spectrometer (MS, Q-Exactive Orbitrap HRMS/MS; Thermo Fisher, Waltham, MA, USA) method was established for metabolomics analysis. The separation column was a UPLC C_18_ column (2.1 × 100 mm, 1.7 μm; ACQUITY BEH, Waters). The composition of the mobile phase was water (A, 0.1% formic acid) and acetonitrile (B). The analysis was carried out with an elution gradient as follows: 0–3 min, 2% B; 3–15 min, 2–100% B; 15–17 min, 100% B. The flow rate was 0.3 mL/min. The column temperature was 35 °C. The injection volume was 3 μL.

MS analysis was performed with a heated electrospray ionization source in both positive and negative ionization modes. The mass parameters were set as follows: auxiliary gas flow, 10 arb; sheath gas flow, 40 arb; spray voltage, 3.5 kV; m/z, 60–900; auxiliary gas heater temperature, 350 °C; and capillary temperature, 320 °C. For multivariate and univariable analysis, the metabolites were searched in the Human Metabolome Database (http://www.hmdb.ca/, accessed on 16 September 2022). MetaboAnalyst (http://www.metaboanalyst.ca/, accessed on 27 December 2022) was used to explore the key metabolic pathways represented by the differential metabolites. The multivariate data matrix was imported into SIMCA 14.0 (Umetrics, Umeå, Sweden) for visualization.

### 2.5. Targeted Metabolomics Analysis

BA contents in the serum, liver, and feces samples were determined using the UPLC-MS (ACQUITY, Waters) with an electrospray negative ionization source. The extraction method was used according to previous reports [18]. Briefly, the liver and feces samples were homogenized in liquid nitrogen and suspended in ultrapure water. A total of 100 μL of each sample or serum was used for analysis. d4-glycocholic acid (d4-GCA), d4-chenodeoxycholic acid (d4-CDCA), d4-cholic acid (d4-CA); d4-glycochenodexycholic acid (d4-GCDCA), and d4-lithocholic acid (d4-LCA) were used as the internal standards. The sample was separated on a UPLC C_18_ (2.1 × 100 mm, 1.7 μm; ACQUITY BEH, Waters). The mobile phase consisted of water (0.1% formic acid, A) and acetonitrile (B). The flow rate was 0.3 mL/min. The analysis was carried out with an elution gradient as follows: 0–2 min, 35% B; 2–10 min, 35–90% B; 10–13 min, 90% B. BAs were detected using the multiple reaction monitoring mode (Table S1, Supplementary Material). MS data were processed using Masslynx V4.1 software (Waters, Beverly, MA, USA).

### 2.6. Western Blotting Analysis

The liver was homogenized and extracted in RIPA lysis buffer (Beyotime, Shanghai, China). The protein content was measured using a BCA kit (Keygentec, Nanjing, China). The primary antibodies included CYP7A1 (#DF2612; Affinity, West Bridgford, UK), CYP27A1 (#DF3571; Affinity), CYP7B1 (#DF3592; Affinity), and CYP8B1 (#DF4762; Affinity). The membrane was incubated with goat anti-rabbit IgG (#BKR050’ Bioker, Hangzhou, China). Protein band densities were analyzed using ImageJ software, latest version Version 1.53t (National Institute of Health, Bethesda, MD, USA).

### 2.7. RNA Isolation and Real-Time Reverse Transcription Polymerase Chain Reaction (RT-PCR)

Total RNA was extracted using TRIzol (Invitrogen, Carlsbad, CA, USA) and reverse transcribed using a TOBOBlue qRT Premix with a gDNA Eraser 2.0 kit (#RTQ202; Toroivd Technology, Shanghai, China). RT-PCR (7500 RT-PCR; Applied Biosystems, Waltham, MA, USA) was used to analyze the levels of CYP7A1, CYP27A1, CYP7B1, and CYP8B1 using SYBR Green reagents (#QPS201; Toyobo, Osaka, Japan). The primer sequences are listed in Table S2. The relative mRNA expression was quantified by comparing cycle threshold values. GAPDH was used as a housekeeping gene. Data are shown as the fold change relative to controls.

### 2.8. 16S rDNA Gene High-Throughput Sequencing

The total bacterial genomic DNA was extracted using a MagaBio Feces Genomic DNA Purification kit (#BSC48; Hangzhou Bioer Technology, Zhejiang, China) according to the manufacturer’s protocol. The quality and quantity of the DNA were analyzed using a microvolume spectrophotometer (NanoDrop One; Thermo Fisher). DNA sequences were amplified using a PCR (S1000; Bio-Rad, Hercules, CA, USA) with primers F515/R806 with a dual indexing approach. A total of 50 μL of PCR reactions was prepared in duplicate and contained 50 ng DNA template, 200 nM primers (Invitrogen, Carlsbad, CA, USA), 2 × Premix Taq (#RR902A; TaKaRa, Dalian, China), and nuclease-free water. Reactions were carried out with an initial denaturation at 94 °C for 5 min, followed by 30 cycles of 30 s at 94 °C, 30 s at 52 °C, 30 s at 72 °C, and then at 72 °C for 10 min. The quality of the pooled sample was evaluated using a high-throughput nucleic acid protein analysis system (Houze Biological Technology, Hangzhou, China) and a Qubit 4.0 Fluorometer (Life Technologies, Grand Island, NY, USA).

Sequence data were processed using an Illumina 6000 platform (Illumina, San Diego, CA, USA). The remaining effective sequences with ≥ 97% similarity were clustered into the same operational taxonomic units (OTUSA) using UPARSE. For each representative sequence, KRONA (http://sourceforge.net/projects/krona/, accessed on 23 July 2022) and GraPhlAn software Version: 1.1.3. (http://huttenhower.sph.harvard.edu/graphlan, accessed on 25 July 2022) were used to annotate taxonomic information. Principal correlation coefficient analysis (PCoA) based on the Bray–Curtis distance was implemented using R programming language (R Foundation for Statistical Computing, Vienna, Austria; https://www.r-project.org/, accessed on 10 August 2022).

### 2.9. Statistical Analysis

Statistical analysis was carried out using GraphPad Prism 7.0 (GraphPad, San Diego, CA, USA). Statistical analysis was conducted using one-way or two-way analysis of variance, followed by Dunnett corrections to compare multiple groups. Data were presented as means ± SD. *p* < 0.05 was considered statistically significant.

## 3. Results

### 3.1. SPN Reduces Hepatic Lipid Accumulation in D-Gal-Induced Mice

**Figure 1:**
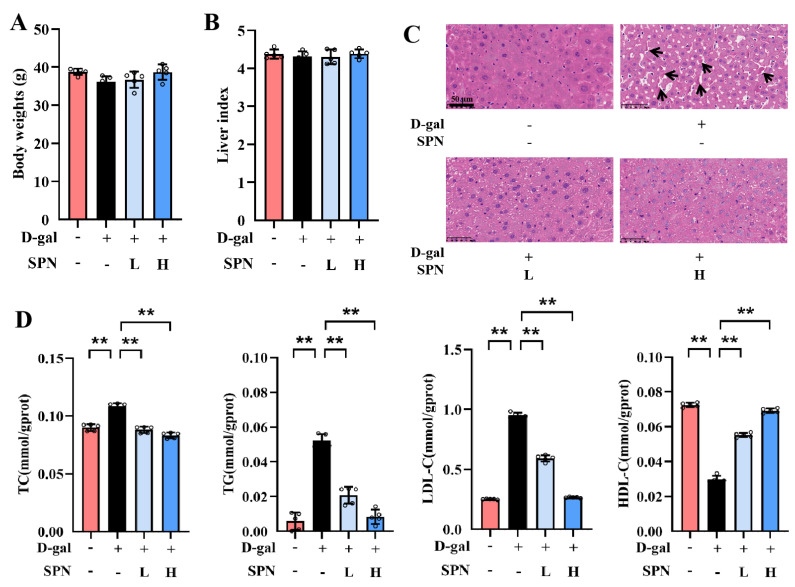
Specnuezhenide (SPN) attenuates age-related hepatic lipid accumulation. (**A**) Body weights. (**B**) The liver index is presented. (**C**) Representative images of liver specimens stained with haematoxylin−eosin staining (H&E) (inflammatory infiltration and focal necrosis, black arrows, scale bar = 50 μm). (**D**) Total cholesterol (TC), total triglyceride (TG), low−density lipoprotein cholesterol (LDL−C), and high−density lipoprotein cholesterol (HDL−C) concentrations in the liver of mice were quantified using the enzymatic kit (*n* = 5). Data are expressed as mean ± SD. ** *p* < 0.01 compared with the model (MOD) group.

After 12 weeks of treatment, there were no significant differences in body weight or liver index among the groups (Figure 1A,B). However, lobular inflammation and focal necrosis were observed in the histopathology of the liver in the MOD group (Figure 1C). SPN treatment could attenuate these injuries in the D-gal–induced liver. Furthermore, the hepatic lipid accumulation was examined by measuring TC, TG, LDL-C, and HDL-C in the liver (Figure 1D). TC, TG, and LDL-C contents in the MOD group increased compared with the control group (*p* < 0.01), and the content of HDL-C in the MOD group decreased (*p* < 0.01). SPN treatments decreased the levels of TC (*p* < 0.01), TG (*p* < 0.01), and LDL-C (*p* < 0.01) but increased the HDL-C contents in the D-gal–induced mice (*p* < 0.01), indicating that SPN could decrease hepatic lipid accumulation in D-gal–induced mice.

### 3.2. SPN Reverses BA Profile in Serum, Liver, and Feces in D-Gal–Induced Mice

**Figure 2:**
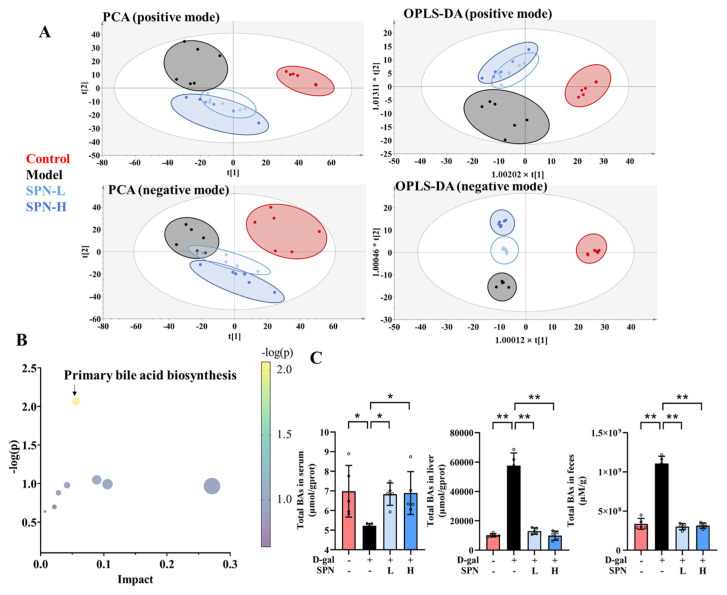
Specnuezhenide (SPN) regulated the metabolic profile and bile acid (BA) compositions. (**A**) The plot depicts separation of the principal component analysis (PCA) and orthogonal partial least-squares discriminant analysis (OPLS−DA) of serum from different groups at positive and negative modes. (**B**) Metabolic pathway analysis of identified potential marker. (**C**) Untargeted metabolomic analyses of serum, liver, and feces were performed to measure the concentration of total BAs (*n* = 5). Data are expressed as mean ± SD. * *p* < 0.05, ** *p* < 0.01 compared with the model (MOD) group.

Metabolomics analysis was carried out to investigate the mechanism underlying the effect of SPN on hepatic lipid accumulation. Untargeted metabolomics data showed that SPN groups were located closer to the CON group in both principal component analysis (Figure 2A, PCA) and orthogonal partial least-squares discriminant analysis (OPLS-DA). These data indicated that the serum metabolic profiles were reversed by SPN treatment. The metabolic pathway enrichment results indicated that primary BA biosynthesis was closely related to the effect of SPN (Figure 2B).

BAs were quantified on the basis of the respective standard curves (Table S3). The total BAs decreased in the serum of the MOD group compared with the CON group (Figure 2C, *p* < 0.05). SPN treatment increased the total BAs in the serum of the D-gal mice (*p* < 0.05). Levels of total BAs in the liver and feces were also reduced in the MOD group (*p* < 0.01). SPN treatment increased the total BAs in the liver of the D-gal mice (*p* < 0.01). Compared with the MOD group, more BAs were excreted through the feces in the SPN-treated mice (*p* < 0.01).

**Figure 3:**
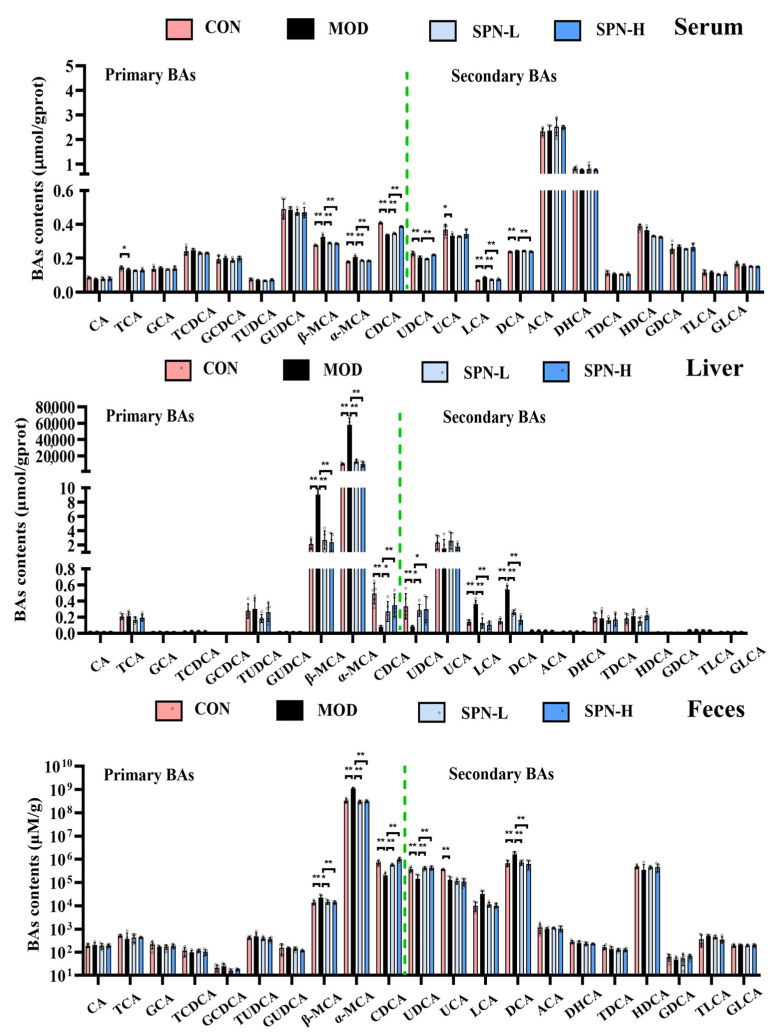
Specnuezhenide (SPN) could regulate bile acid (BA) homeostasis. Targeted metabolomic analyses of serum, liver, and feces samples were performed using UPLC−MS to measure the concentration of individual BA (*n* = 5). Data are expressed as mean ± SD. * *p* < 0.05, ** *p* < 0.01 compared with the model (MOD) group.

We compared the contents of individual BAs in the serum, liver, and feces from different groups. Several BAs in the serum from the MOD group were significantly reduced, including taurocholic acid (TCA, Figure 3, *p* < 0.05), chenodeoxycholic acid (CDCA, *p* < 0.01), ursodeoxycholic acid (UDCA, *p* < 0.01), and ursocholic acid (UCA, *p* < 0.05). Meanwhile, the contents of β-muricholic acid (β-MCA, *p* < 0.01), α-muricholic acid (α-MCA, *p* < 0.01), lithocholic acid (LCA, *p* < 0.01), and deoxycholic acid (DCA, *p* < 0.01) in the MOD group were higher than those in the CON group. SPN increased CDCA (*p* < 0.01) and UDCA (*p* < 0.01 for SPN-H) and reduced β-MCA (*p* < 0.01), α-MCA (*p* < 0.01), LCA (*p* < 0.01), and DCA (*p* < 0.01 for SPN-H) compared with those in the MOD group. The changes of CDCA, UDCA, β-MCA, α-MCA, and DCA in the liver and feces were consistent with those in the serum samples. These findings indicated that SPN reversed the D-gal–induced changes of BA compositions in the serum, liver, and feces samples.

### 3.3. SPN Enhanced Bile Acid Synthesis by Stimulating Hepatic Enzymes

**Figure 4:**
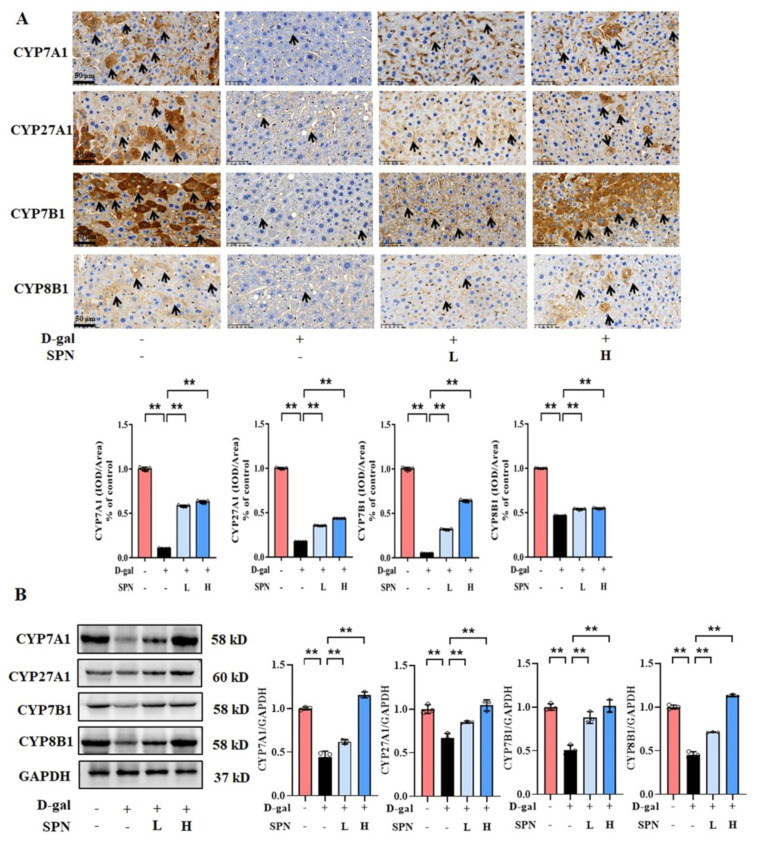
Specnuezhenide (SPN) upregulates the protein expression of bile acid (BA) enzymes in D−galactose (D−gal) mice. (**A**) Immunohistochemistry (IHC) analysis of cytochrome P4507A1 (CYP7A1), cytochrome P45027A1 (CYP27A1), cytochrome P4507B1 (CYP7B1), and cytochrome P4508B1 (CYP8B1) in the liver (black arrows, *n* = 5). (**B**) The levels of CYP7A1, CYP27A1, CYP7B1, and CYP8B1 were analyzed using western blotting. The densitometric quantification of CYP7A1, CYP27A1, CYP7B1, and CYP8B1 is shown in the right panels (*n* = 3). Data are expressed as mean ± SD. ** *p* < 0.01 compared with the model (MOD) group.

**Figure 5:**
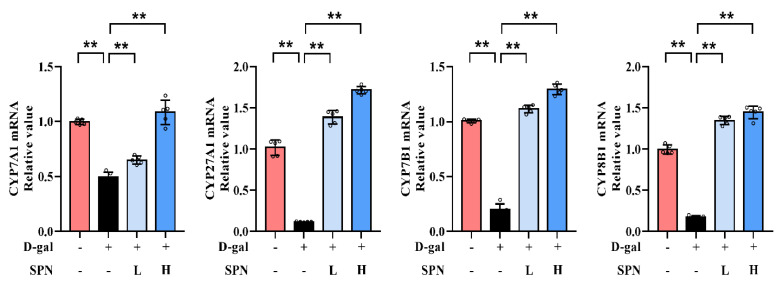
Specnuezhenide (SPN) upregulates the mRNA expressions of cytochrome P4507A1 (CYP7A1), cytochrome P45027A1 (CYP27A1), cytochrome P4507B1 (CYP7B1), and cytochrome P4508B1 (CYP8B1) mRNA in the liver (*n* = 5). ** *p* < 0.01 compared with the model (MOD) group.

Since primary BA synthesis occurs in the liver, we examined the effect of SPN on the hepatic enzymes for bile acid synthesis, including CYP7A1, CYP7B1, CYP8B1, and CYP27A1. IHC results showed that the protein levels of CYP7A1 (Figure 4A, *p* < 0.01), CYP7B1 (*p* < 0.01), CYP8B1 (*p* < 0.01), and CYP27A1 (*p* < 0.01) were downregulated in the MOD group compared to the CON group, while SPN treatment restored these changes (*p* < 0.01). The expressions of CYP7A1, CYP7B1, CYP8B1, and CYP27A1 in the liver were also analyzed using western blotting (Figure 4B) and RT-PCR (Figure 5). The levels of these enzymes decreased in the MOD group compared with the CON group (*p* < 0.01), which was consistent with a previous report [19]. Importantly, the expression levels of CYP7A1 (*p* < 0.01), CYP7B1 (*p* < 0.01), CYP8B1 (*p* < 0.01), and CYP27A1 (*p* < 0.01) in the D-gal–induced livers were reversed in response to the SPN treatment. The above results suggested that SPN activated both the classical and alternative pathways of BA synthesis.

### 3.4. SPN Remodels the Gut Microbiota Genera Associated with Bile Salt Hydrolases

**Figure 6:**
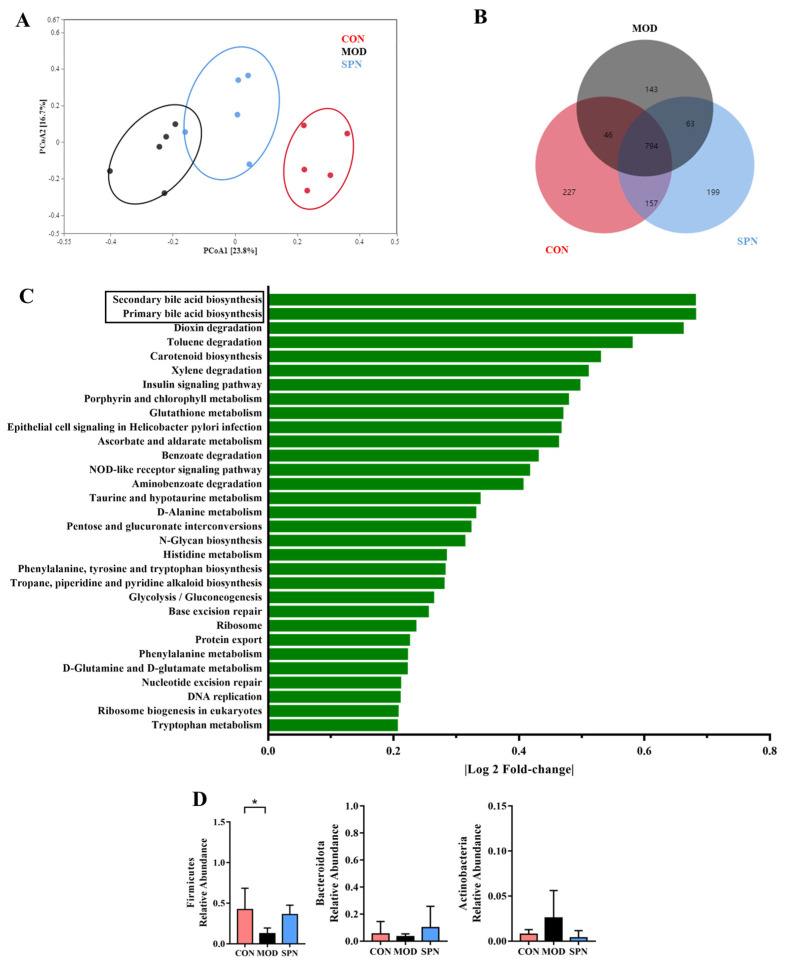
Specnuezhenide (SPN) treatment improved gut microbiota disbalance. (**A**) Principal correlation coefficient analysis (PCoA) analysis of gut microbiota at operational taxonomic units (OTUSA) level based on Bray−Curtis. (**B**) Shared and unique OTUs among the different groups. (**C**) The predicted functional metabolic pathways. (**D**) The relative abundance of bile salt hydrolase (BSH) related phylum (*n* = 5). Data are expressed as mean ± SD. * *p* < 0.05 compared with the MOD group.

Given the critical crosstalk between the gut microbiota and BA metabolism, 16S rDNA high-throughput sequencing of the feces was used to evaluate the effect of SPN on gut microbiota. The Bray–Curtis PCoA revealed a total separation between the bacterial profiles of the CON, MOD, and SPN-H groups (Figure 6A). These groups shared 794 common OTUs (Figure 6B). Among the predicted functional metabolic pathways (Figure 6C), secondary BA biosynthesis and primary BA biosynthesis were affected by gut microbiota.

Bile salt hydrolase (BSH) was found in three phyla in D-gal–induced mice, including *Firmicutes*, *Bacteroidetes,* and *Actinobacteria.* D-gal injection significantly reduced *Firmicutes* (Figure 6D). There was no significant difference in the *Firmicutes*-to-*Bacteroides* ratio or α-diversity indices between groups (Figure S1). The relative abundance of the family *Lactobacillaceae* from *Firmicutes* displayed different profiles in the MOD and CON groups (Figure S2). Specifically, SPN increased the abundance of the BSH-related genera *Lactobacillus* from *Lactobacillaceae* (Figure S3, *p* < 0.05). *Lactobacillus* might contain BSH-T0, BSH-T2, and BSH-T3 [20]. Additionally, SPN upregulated the abundance of *Ruminiclostridium* (*p* < 0.01) and *Butyrivibrio* (*p* < 0.01), which might contain BSH-T. These data indicated that SPN could affect BA compositions by regulating gut microbiota in D-gal mice.

## 4. Discussion

Hepatic lipid accumulation implies injury to the liver and most likely causes NAFLD in elderly populations. Recently, natural hepatoprotective compounds have attracted increasing attention. SPN is a major active component from an edible herb *Fructus Ligustri Lucidi*, which can tonify the liver. However, the protective effects of SPN against age-related liver injury remain unclear. In this study, SPN treatment could attenuate the hepatic lipid accumulation and inflammation in D-gal–induced mice. Untargeted and targeted metabolomics analysis proved that the BA synthesis pathway was crucial for the protection effects of SPN. SPN treatment restored BA compositions in the serum, liver, and feces of D-gal–induced mice. Furthermore, we found that SPN could upregulate the expressions of the hepatic BAs synthesis enzymes and modulate the gut microbiota.

Mass spectrometry is a powerful technique for investigating metabolic features associated with aging [21]. A non-targeted analysis of human blood metabolome disclosed a close link between metabolites and aging [22]. Consistent with this, our untargeted metabolomics revealed that shifts in metabolomics profiles were observed in D-gal–induced mice. Treatment with SPN moderately corrected this metabolic change. An altered BA profile is tightly related to the development of age-related liver disease [23]. The enrichment analysis showed that primary BA biosynthesis was the most significant metabolic pathway in response to the SPN treatment. BA metabolism is a promising target for developing antisenescence-based therapies. We established a method to simultaneously quantify 21 BAs in the serum, liver, and feces. Targeted-BA metabolomics demonstrated that changes in serum BA contents appeared in response to D-gal injection. SPN restored six BAs in the D-gal–induced mice. Our data showed that SPN treatment increased CDCA in the D-gal-induced mice. Additionally, NAFLD is characterized by increased DCA and LCA [24]. Excessive DCA produces reactive oxygen species that cause DNA damage and senescence-associated secretory phenotypes, inducing a pro-inflammatory response in steatosis hepatocytes [25], which facilitates the development of aging-related liver disease [26]. Additionally, LCA shows a deleterious effect on the liver [27], but these effects can be neutralized by UDCA [28]. In our study, SPN reduced the contents of DCA and LCA in the serum, liver, and feces of the D-gal–induced mice. These results indicated that SPN may attenuate D-gal–induced hepatic lipid accumulation and injury via two pathways: (1) increasing the CDCA and UDCA content, which is positively related to cholesterol absorption [29]; (2) reducing the DCA level, which contributes to hepatotoxicity in the mice [30].

BAs are synthesized as primary BAs in the liver through enzymatic pathways [31]. Cholesterol is hydroxylated in the 7α-position by CYP7A1 or converted to an oxysterol before being in the 7α-position by CYP7B1 and arises in the liver by CYP27A. However, liver function deteriorates with age, leading to downregulated BA synthesis enzymes and reduced BA amount [32]. In our study, D-gal injection reduced the expression of CYP7A1, CYP27A1, CYP7B1, and CYP8B1 in mouse liver. The pathophysiological changes of these BA synthesis enzymes are risk factors for the development of lipid accumulation and cholesterol metabolism [33]. Iridoid glycosides are an important source of BA synthesis modulators. For example, swertiamarin [34] and gentiopicroside [35] showed hepatoprotective effects in alpha-naphthylisothiocyanate–induced mice via upregulating CYP8B1 and CYP27A1. Our data showed that SPN enhanced the expression of these four key BAs enzymes (CYP7A1, CYP27A1, CYP7B1, and CYP8B1) in the liver. These findings suggest that SPN promoted BA synthesis by stimulating the hepatic BA synthesis enzymes in D-gal–induced mice.

Furthermore, primary BAs are modified in the intestine through the actions of bacteria, such as deconjugation via BSH activity [36]. Since the abundance and diversity of the gut microbiota in mice are changed upon senescence [37], the BA composition also changes during aging. In our study, the age-related model is commonly established by D-gal injection. The action of D-gal–induced models is largely attributed to changes in gut microbial populations [38]. In the present study, D-gal injection changed gut microbiota compositions with BSHs, which has essential effects on secondary BA production. A very intriguing finding was that SPN could reverse three BSH-related genera, including *Lactobacillus*, *Ruminiclostridium*, and *Butyrivibrio*. The decreased level of *Lactobacillus* results in increased levels of Tauro-β-MCA and a substrate of BSHs [39]. *Ruminiclostridium* and *Butyrivibrio* might contain BSH-T1, and BSH-T1 exerted vigorous enzyme activity when the substrates were TCDCA and GCDCA, resulting in a significant change in CDCA content [40]. Our data indicated that SPN regulated the relative abundance of BAs at least partially by increasing the gut microbes that possess BSHs.

## 5. Conclusions

In this work, SPN, one of the iridoid glucoside representatives, the most potential active component in *Fructus Ligustri Lucidi*, could attenuate D-gal–induced hepatic lipid accumulation and injury through regulating the expression of BA synthesis enzymes and the structure of the gut microbiota. Our study provides a theoretical basis that SPN may be a functional factor supplement for treating age-related hepatic lipid accumulation via modulation of BA homeostasis. Future work may identify the toxicity and the clinical application of this compound.

## Data Availability

Data is not publicly available due to privacy or ethical restrictions. The data that support the findings of this study are available from the corresponding author upon reasonable request.

## Supplementary Materials

The following supporting information can be downloaded at https://www.mdpi.com/article/10.3390/metabo13080960/s1, Table S1: MRM parameters for bile acids analysis; Table S2: Primer sequences for RT-PCR; Table S3: Linear equation and limit of detection (LOD) of bile acid in serum, liver and fecse; Figure S1: Microbial richness and diversity of different groups based on the Chao1 index and the Shannon index; Figure S2: The relative abundance of OTUs at the family levels in the gut microbiota (*n* = 5); Figure S3: The relative abundance of OTUs at the family levels in the gut microbiota (*n* = 5).
