# Supplementary material for: Specnuezhenide Ameliorates Age-Related Hepatic Lipid Accumulation via Modulating Bile Acid Homeostasis and Gut Microbiota in D-Galactose-Induced Mice
Source: Metabolites. 2023 Aug 18;13(8):960. doi: 10.3390/metabo13080960 (PMC10456809; doi:10.3390/metabo13080960)
Supplement: Supplementary file 1 [file metabolites-13-00960-s001.zip › metabolites-2470169-supplementary.pdf]

metabolites

Supplementary Materials

Table S1. MRM parameters for bile acids analysis.

Compound	Abbreviation	Transition	Cone	Collision
Cholic acid	CA	407.3 > 343.3	160	36
Taurocholic acid	TCA	514.3 > 80.0	150	110
Glycocholic acid	GCA	464.3 > 74.1	125	36
Taurochenodexycholic acid	TCDCA	498.3 > 80.0	130	115
Glycochenodexycholic acid	GCDCA	448.2 > 74.1	125	30
Tauroursodeoxycholic acid	TUDCA	498.3 > 80.0	120	75
Glycoursodeoxycholic acid	GUDCA	448.2 > 74.1	120	50
β-muricholic acid	β-MCA	407.3 > 407.3	160	10
α-muricholic acid	α-MCA	407.3 > 407.3	150	20
Chenodeoxycholic acid	CDCA	391.3 > 391.3	145	15
Ursodeoxycholic acid	UDCA	391.3 > 391.3	155	15
Ursocholic acid	UCA	407.3 > 407.3	150	20
Lithocholic acid	LCA	375.2 > 375.2	150	10
Deoxycholic acid	DCA	391.3 > 391.3	145	10
Allocholic acid	ACA	175.1 > 175.1	150	40
Dehydrocholic acid	DHCA	401.1 > 249.2	120	40
Taurodeoxycholic acid	TDCA	498.3 > 80.0	140	105
Hyodeoxycholic acid	HDCA	391.3 > 391.3	150	10
Glycodeoxycholic acid	GDCA	448.3 > 74.1	125	42
Taurolithocholic acid	TLCA	482.3 > 80	150	110
Glycolithocholic acid	GLCA	432.4 > 74.1	120	68

Table S2. Primer sequences for RT-PCR.

Gene	Forward (5′-3′)	Reverse (5′-3′)
CYP7A1	AGGTCTCTGAACTGATCCGTCTACG	GCGTCTTAGCCTTCTCCATGTCATC
CYP27A1	CACCGATGGCTGAGGAAGAAAGAG	ACCCAGGCAAGACCGAACCC
CYP7B1	GCCTCTCTAGCAAACACCATTCCAG	AATTTCGTCACGCAGGGCTTCC
CYP8B1	ACACCAAGGACAAGCAGCAAGAC	TGGCTCACTTCCACCCACTCC
GAPDH	GGCAAATTCAACGGCACAGTCAAG	TCGCTCCTGGAAGATGGTGATGG

Table S3. Linear equation and limit of detection (LOD) of bile acid in serum, liver and feces.

Compound	Linear Equation	SE (Slope / Intercept)	R^2	LOD (μmol/L)
CA	y = 31.39x − 10.11	0.05 / 0.06	0.994	2.94
TCA	y = 33.48x − 11.16	0.05 / 0.07	0.9999	2.62
GCA	y = 13.36x − 3.79	0.04 / 0.06	0.9998	2.30
TCDCA	y = 31.95x − 5.50	0.04 / 0.06	0.9994	2.62
GCDCA	y = 12.95x − 0.11	0.11 / 0.05	0.9999	1.83
TUDCA	y = 10.11x − 3.35	0.08 / 0.06	0.996	1.43
GUDCA	y = 25.48x − 7.24	0.14 / 0.11	0.998	3.02
β-MCA	y = 7.359x − 1.24	0.07 / 0.04	0.992	1.90
α-MCA	y = 6.714x − 0.91	0.04 / 0.05	0.9992	1.90
CDCA	y = 8.808x + 0.33	0.06 / 0.06	0.995	3.25
UDCA	y = 13.56x − 2.18	0.08 / 0.03	0.997	2.86
UCA	y = 8.968x − 1.31	0.06 / 0.06	0.9999	2.30
LCA	y = 9.703x − 2.14	0.08 / 0.04	0.998	2.14
DCA	y = 5.911x + 0.98	0.06 / 0.04	0.997	2.86
ACA	y = 244.9x − 96.19	0.06 / 0.06	0.9994	4.52
DHCA	y = 50.27x − 16.32	0.04 / 0.13	0.991	3.33
TDCA	y = 7.098x − 1.07	0.07 / 0.05	0.9995	2.30
HDCA	y = 6.059x + 2.08	0.05 / 0.12	0.9994	3.25
GDCA	y = 4.240x + 0.03	0.08 / 0.01	0.9996	2.38
TLCA	y = 7.778x −5.93	0.02 / 0.07	0.993	2.22
GLCA	y = 8.592x − 2.05	0.03 / 0.06	0.992	1.43

Note: Correlation Coefficient (R^2), Standard Error (SE).

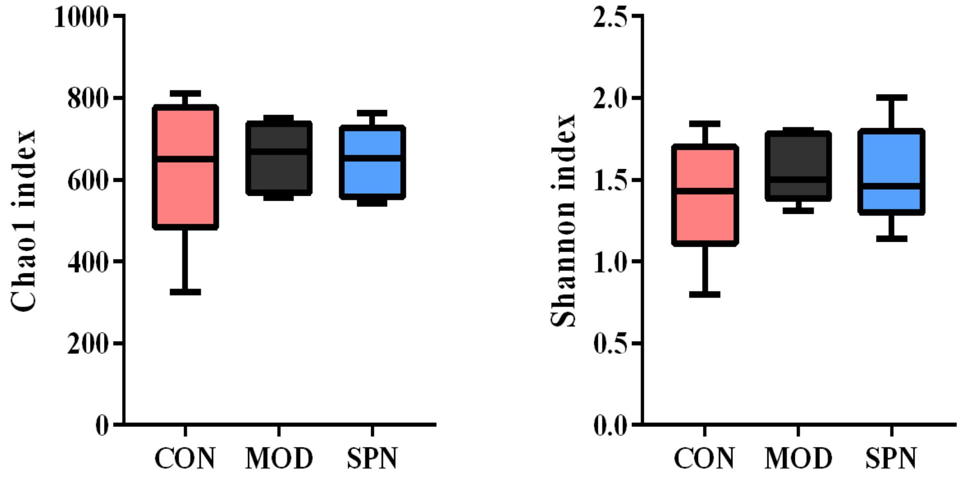

Figure S1. Microbial richness and diversity of different groups based on the Chao1 index and the Shannon index.

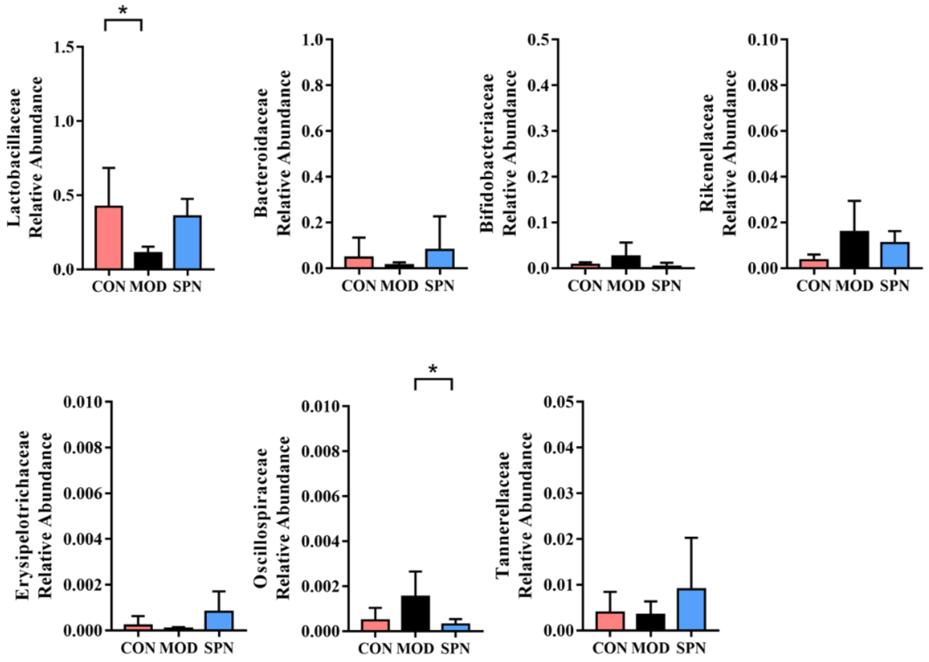

Figure S2. The relative abundance of OTUs at the family levels in the gut microbiota (n = 5). Data are expressed as the mean ± SD. *: $P < 0.05$ compared with the MOD group.

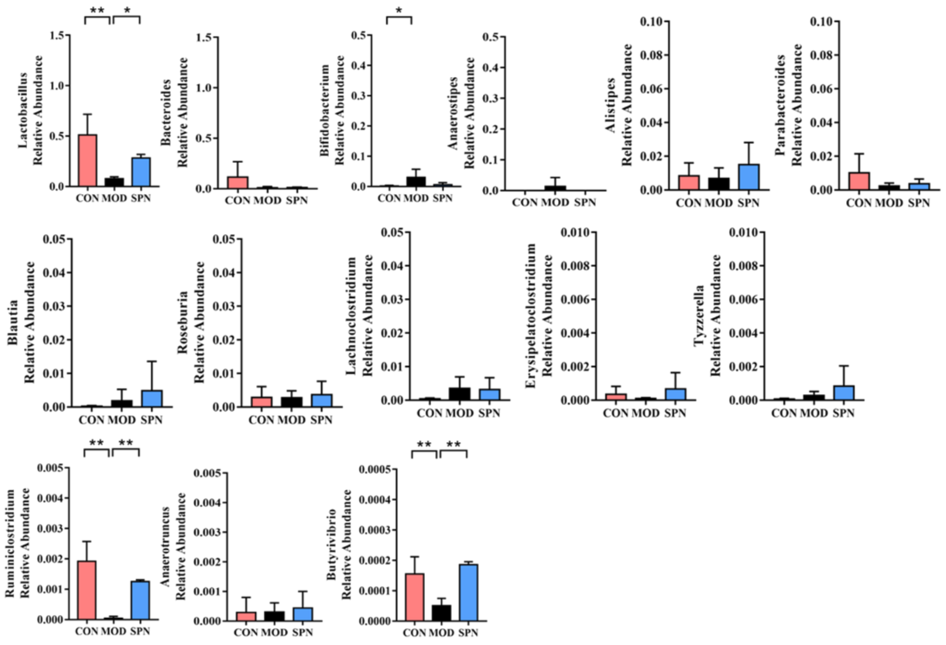

Figure S3. The relative abundance of OTUs at the genera levels in the gut microbiota (n = 5). Data are expressed as the mean ± SD. *: $P < 0.05$, **: $P < 0.01$ compared with the MOD group.
